# Channels with ordered water and bipyridine mol­ecules in the porous coordination polymer {[Cu(SiF_6_)(C_10_H_8_N_2_)_2_]·2C_10_N_2_H_8_·5H_2_O}_*n*_

**DOI:** 10.1107/S2056989016016686

**Published:** 2016-10-25

**Authors:** Emmanuel Aubert, Abdelatif Doudouh, Paola Peluso, Victor Mamane

**Affiliations:** a Cristallographie, Résonance Magnétique et Modélisations (CRM2), UMR CNRS, 7036, Université de Lorraine, BP 70239, Bd des Aiguillettes, 54506, Vandoeuvre-les-Nancy, France; b Istituto di Chimica Biomolecolare ICB CNR - Sede Secondaria di Sassari, Traversa La Crucca 3, Regione Baldinca, I-07100 Li Punti - Sassari, Italy; c Institut de Chimie de Strasbourg, UMR 7177, Equipe LASYROC, 1 rue Blaise Pascal, BP 296 R8, 67008 Strasbourg Cedex, France

**Keywords:** Porous coordination polymer, adsorption, hydrogen bonding, π–π stacking, copper(II), 4,4′-bi­pyridine, crystal structure

## Abstract

The coordination polymer {[Cu(SiF_6_)(C_10_H_8_N_2_)_2_]·2C_10_H_8_N_2_·5H_2_O}_*n*_, systematic name: poly[[bis­(μ_2_-4,4′-bi­pyridine)(μ_2_-hexa­fluorido­silicato)copper(II)] 4,4′-bi­pyridine disolvate penta­hydrate], contains pores which are filled with water and 4,4′-bi­pyridine mol­ecules. As a result of the presence of these ordered species, the framework changes its symmetry from *P*4/*mmm* to *P*2_1_/*c*. The 4,4′-bi­pyridine guest mol­ecules form chains inside the 6.5 × 6.9 Å pores parallel to [100] in which the mol­ecules inter­act through π–π stacking. Ordered water mol­ecules form infinite hydrogen-bonded chains inside a second pore system (1.6 × 5.3 Å free aperture) perpendicular to the 4,4′-bi­pyridine channels.

## Abstract 2

The structure of a [Cu(SiF_6_)(C_10_H_8_N_2_)_2_]_*n*_ coordination polymer with ordered 4,4′-bi­pyridine and water mol­ecule channels is described.

## Chemical context   

The title compound was obtained in an attempt to reproduce the synthesis of [Cu(μ-4,4′-bipy)(H_2_O)_2_(BF_4_)_2_]·4,4′-bipy (Blake *et al.*, 1997 ▸). A contamination with SiF_6_^2−^ is, however, at the origin of the formation of {[Cu(SiF_6_)(C_10_H_8_N_2_)_2_]·2C_10_N_2_H_8_·5H_2_O}_*n*_, whose framework was previously described by Noro *et al.* (2000 ▸, 2002 ▸). This framework has shown inter­esting gas adsorption properties in recent years (Burd *et al.*, 2012 ▸; Yu *et al.*, 2012 ▸; Fan *et al.*, 2013 ▸). Several structures based on this porous framework have been published since its discovery [CSD refcodes: GORWUF (Noro *et al.*, 2000 ▸), AFEKAX (Noro *et al.*, 2002 ▸), HAPKOA (Burd *et al.*, 2012 ▸)]. However, these structures which are reported in the tetra­g­onal space group type *P*4/*mmm* are disordered: the framework bi­pyridines are disordered by symmetry whereas solvent mol­ecules are not clearly identified within the pores. In this article, we show that this porous coordination polymer is capable of firmly stabilizing guest entities such as 4,4′-bi­pyridine and water mol­ecules within its channels. The synthesis conditions thus seem a key factor in producing the ordering of guest mol­ecules within this porous material.

## Structural commentary   

**Figure 1:**
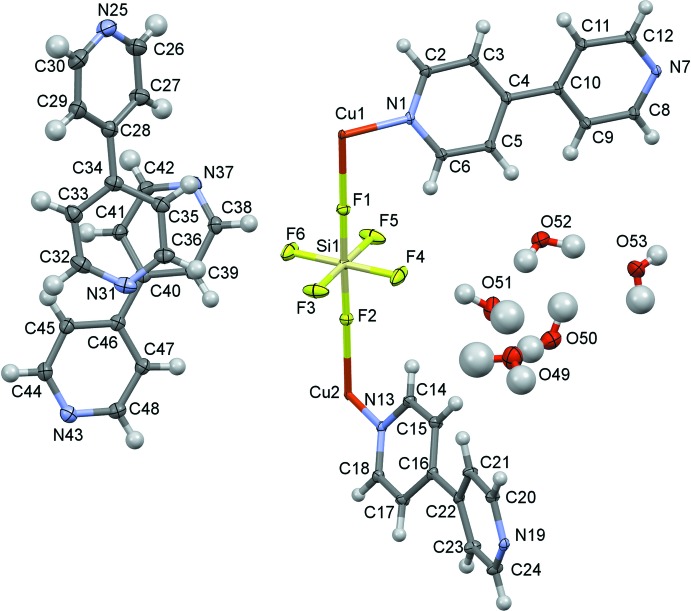
The asymmetric unit of the title compound. Displacement ellipsoids are drawn at the 50% probability level.

The asymmetric unit of the title compound (Fig. 1 ▸) contains two copper(II) atoms, both lying on inversion centers; each of these two atoms is coordinated by N atoms of four symmetrically related 4,4′-bi­pyridine mol­ecules (with one independent bi­pyridine for each copper atom), forming slightly distorted two-dimensional square grids parallel to (100). The copper(II) atoms are both at the center of elongated octa­hedra (Table 1 ▸).
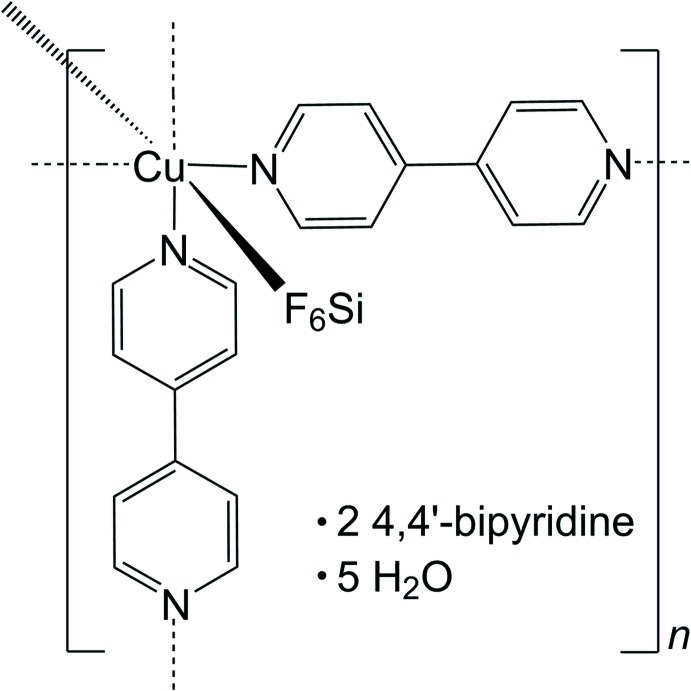


**Figure 2:**
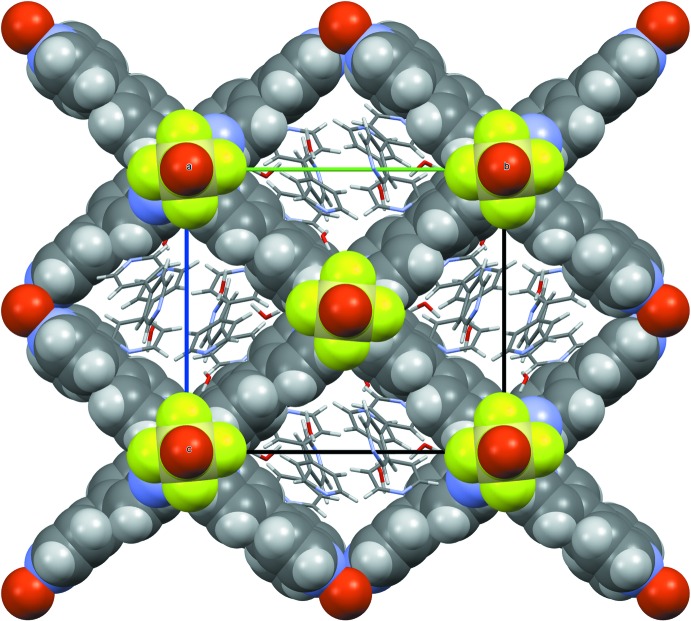
View along [011]; the atoms belonging to the framework are shown as space-filling, whereas the mol­ecules adsorbed inside the pores are shown as capped sticks.

The basal plane is composed of four nitro­gen atoms coming from the 4,4′-bi­pyridine mol­ecules, whereas the apical positions are occupied by fluorine atoms belonging to the SiF_6_^2−^ anions pillaring the structure (Fig. 2 ▸).

**Figure 3:**
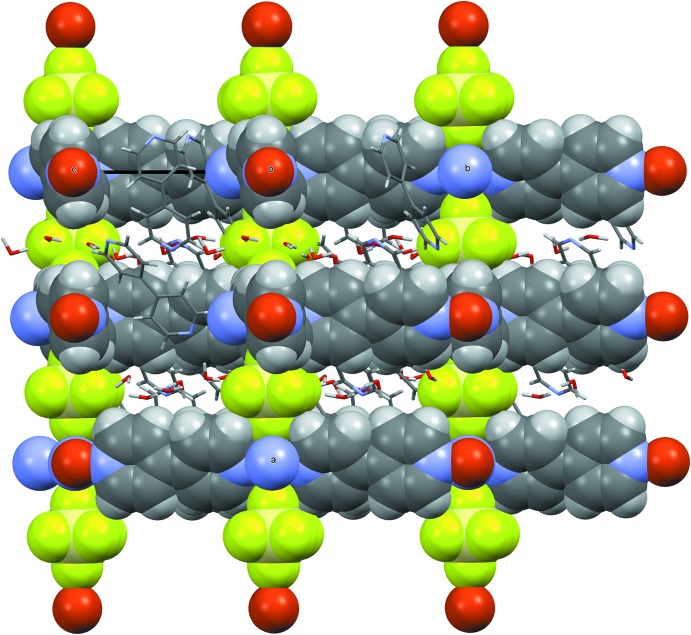
View along [100]; the atoms belonging to the framework are shown as space-filling, whereas the mol­ecules inside the pores are shown as capped sticks.

The 2D coordination grids are stacked along the [100] direction through the SiF_6_^2−^ anions, leading to a three-dimensional coordination polymer, which displays channels having a free aperture of 6.5 × 6.9 Å parallel to [100] and smaller pores of 1.6 × 5.3 Å along the [011] direction (as measured in projection in the plane perpendicular to the channels and using van der Waals radii). These inter­connected pores are filled with two other 4,4′-bi­pyridine mol­ecules and five water mol­ecules (Figs. 3 ▸ and 4 ▸). In comparison, the previously reported structures with this framework are described in the *P*4/*mmm* space group type, implying a squared Cu grid and channels; here, the Cu–Cu–Cu grid angle significantly deviates from 90° (96.62°) and this may be related to the fact that, in the present compound, guest mol­ecules fill the pores and inter­act significantly with the framework atoms (see below).

The Si—F bond lengths in SiF_6_^2−^ show some variations (Table 1 ▸), ranging from 1.6534 (11) to 1.7145 (10) Å. The two longest bond lengths are associated with opposite fluorine atoms bounded to a Cu^II^ metal atom. Among the four remaining fluorine atoms, three of them form short hydrogen bonds with water mol­ecules and display longer bond lengths than the last one, which only forms a weaker hydrogen-bonding inter­action with a 4,4′-bi­pyridine mol­ecule (Table 2 ▸). A search for SiF_6_^2−^ anions within the Cambridge Structural Database (CSD Version 5.36; Groom *et al.*, 2016 ▸) leads to 241 hits (using options ‘not disordered’" and ‘no errors’); the reported Si—F bond lengths range from 1.577 to 1.748 Å with a mean of 1.684 Å and a standard deviation of 0.022 Å.

## Supra­molecular features   

**Figure 5:**
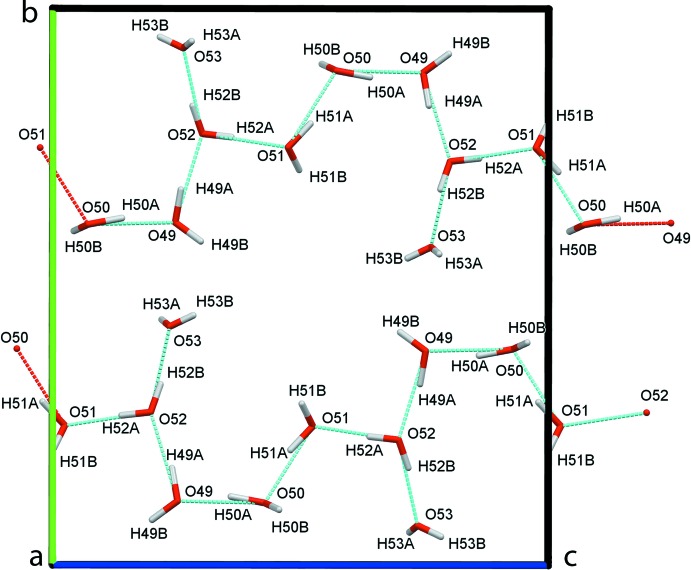
Hydrogen-bonding pattern of solvate water mol­ecules, forming infinite chains along the [001] direction. All other atoms apart from those of the water mol­ecules are omitted for clarity.

**Figure 6:**
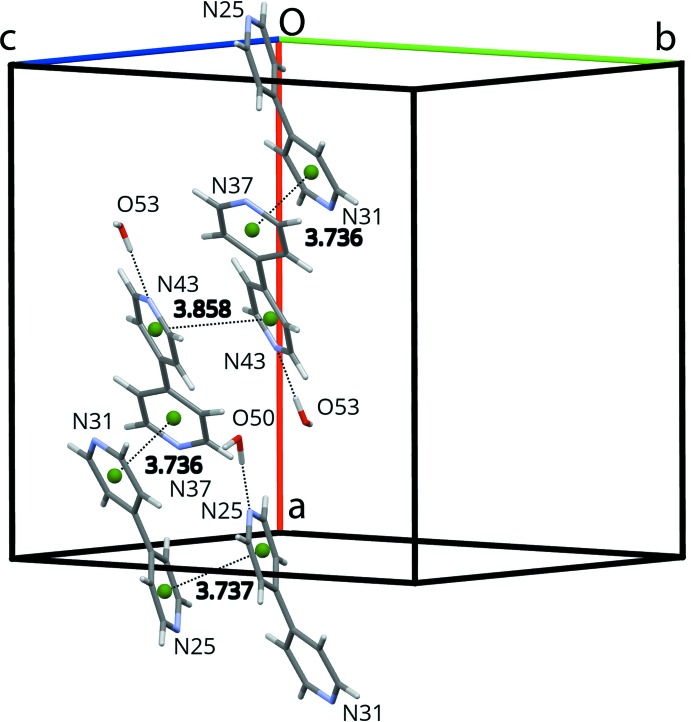
π–π stacking pattern of the solvent 4,4′-bi­pyridine mol­ecules in the [100] pores. All other atoms (except water mol­ecules hydrogen-bonded to N atoms of these bi­pyridines) are omitted for clarity. Pyridyl centroids are displayed as green spheres.

Four of the five water mol­ecules (O49 to O52) form infinite 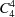
(2) chains running in the [001] direction throughout the pores (Fig. 5 ▸). The fifth water mol­ecule (O53) inter­acts with these chains and several hydrogen bonds anchor these water mol­ecules to the coordination polymer framework (Table 2 ▸). The 4,4′-bi­pyridine mol­ecules filling the [100] channels form chains through π–π stacking (Fig. 6 ▸; Table 3 ▸), and are connected to three of the water mol­ecules (O50, O51 and O53) and framework fluorine and aromatic hydrogen atoms by hydrogen bonds (Table 2 ▸); these inter­molecular inter­actions induce different dihedral angles within the two symmetry-independent 4,4′-bi­pyridine mol­ecules [bipy_(N25–N31)_: 45.29 (7)°; bipy_(N37–N43)_: 30.31 (7)°]. Whereas the 4,4′-bi­pyridine mol­ecules belonging to the coordination network are rather rigid between the metal atoms [average *U*_eq_ = 0.014 (2) Å^2^ as calculated on C,N atoms], the adsorbed 4,4′-bi­pyridine mol­ecules display significantly larger atomic displacement parameters [*U*_eq_ = 0.025 (5) Å^2^].

## Database survey   

A survey was performed in the Cambridge Structural Database (CSD Version 5.36; Groom *et al.*, 2016 ▸). Beside the structures corresponding to the bare or hydrated [Cu(SiF_6_)(C_10_H_8_N_2_)_2_]_*n*_ coordination polymer framework [CSD refcodes: GORWUF (Noro *et al.*, 2000 ▸), AFEKAX (Noro *et al.*, 2002 ▸), HAPKOA (Burd *et al.*, 2012 ▸)], several structures related to the title compound have been described. In particular, Noro *et al.* showed that the hydrated form of the title compound {[Cu(SiF_6_)(4,4′-bpy)_2_]·8H_2_O}_*n*_ undergoes a structural conversion when immersed in water, leading to an inter­penetrated network where SiF_6_^2−^ anions are shifted out of the coordination sphere of copper ions and are replaced by water mol­ecules [CSD refcodes: AFEHOI (Noro *et al.*, 2002 ▸); JEZRUB (Gable *et al.*, 1990 ▸)]. When copper is replaced by zinc, an isostructural compound is obtained [CSD refcodes: WONZIJ (Lin *et al.*, 2009 ▸); ZESFUY (Subramanian & Zaworotko, 1995 ▸)].

## Synthesis and crystallization   

An aqueous solution (5 cm^3^) of hydrated copper(II) tetra­fluorido­borate (47.43 mg, 0.2 mmol) was added to a refluxing aceto­nitrile solution (5 cm^3^) of 4,4′-bi­pyridine (62.48 mg, 0.4 mmol). After filtration, Et_2_O vapor was diffused into the mother liquor for seven days, and then the solvent was allowed to evaporate very slowly. A mixture of blue and violet crystals was obtained; whereas the diffraction spots of the blue crystals could not be properly indexed, the violet crystals were of very good quality and led to the structure reported on herein.

## Refinement   

Crystal data, data collection and structure refinement details are summarized in Table 4 ▸. All hydrogen atoms of water mol­ecules were freely refined in an isotropic approximation. Aromatic hydrogen atoms were refined with riding coordin­ates and *U*_iso_(H) = 1.2*U*_iso_(C).

## Supplementary Material

Crystal structure: contains datablock(s) I. DOI: 10.1107/S2056989016016686/vn2118sup1.cif

Structure factors: contains datablock(s) I. DOI: 10.1107/S2056989016016686/vn2118Isup2.hkl

CCDC reference: 1510381

Additional supporting information: crystallographic information; 3D view; checkCIF report

## Figures and Tables

**Figure 4:**
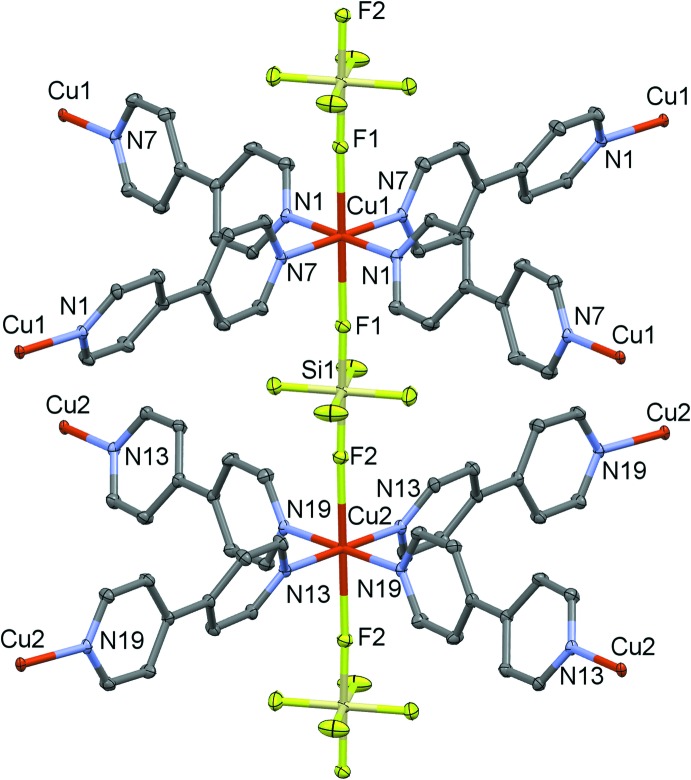
*ORTEP-*style plot of the title compound showing the coordination about the two inequivalent copper atoms. Hydrogen atoms, adsorbed 4,4′-bi­pyridine and water mol­ecules are omitted for clarity. Displacement ellipsoids are drawn at the 50% probability level.

**Table 1:** Selected geometric parameters (Å, °)

Cu1—N1^i^	2.0156 (11)	Cu2—N19^vi^	2.0494 (11)
Cu1—N1	2.0156 (11)	Cu2—F2	2.4109 (9)
Cu1—N7^ii^	2.0467 (11)	Cu2—F2^iv^	2.4109 (9)
Cu1—N7^iii^	2.0467 (11)	Si1—F6	1.6534 (11)
Cu1—F1^i^	2.3585 (9)	Si1—F5	1.6806 (10)
Cu1—F1	2.3585 (9)	Si1—F3	1.6823 (10)
Cu2—N13^iv^	2.0170 (11)	Si1—F4	1.6855 (11)
Cu2—N13	2.0170 (11)	Si1—F2	1.7120 (10)
Cu2—N19^v^	2.0494 (11)	Si1—F1	1.7145 (10)
			
N1^i^—Cu1—N1	180.00 (6)	N19^v^—Cu2—F2	90.67 (4)
N1^i^—Cu1—N7^ii^	91.58 (5)	N19^vi^—Cu2—F2	89.33 (4)
N1—Cu1—N7^ii^	88.42 (5)	N13^iv^—Cu2—F2^iv^	89.41 (4)
N1^i^—Cu1—N7^iii^	88.42 (5)	N13—Cu2—F2^iv^	90.59 (4)
N1—Cu1—N7^iii^	91.58 (5)	N19^v^—Cu2—F2^iv^	89.33 (4)
N7^ii^—Cu1—N7^iii^	180.0	N19^vi^—Cu2—F2^iv^	90.67 (4)
N1^i^—Cu1—F1^i^	90.65 (4)	F2—Cu2—F2^iv^	180.0
N1—Cu1—F1^i^	89.35 (4)	F6—Si1—F5	90.80 (6)
N7^ii^—Cu1—F1^i^	89.60 (4)	F6—Si1—F3	89.64 (6)
N7^iii^—Cu1—F1^i^	90.40 (4)	F5—Si1—F3	179.55 (6)
N1^i^—Cu1—F1	89.35 (4)	F6—Si1—F4	178.87 (6)
N1—Cu1—F1	90.65 (4)	F5—Si1—F4	90.12 (6)
N7^ii^—Cu1—F1	90.40 (4)	F3—Si1—F4	89.44 (6)
N7^iii^—Cu1—F1	89.60 (4)	F6—Si1—F2	91.01 (4)
F1^i^—Cu1—F1	180.0	F5—Si1—F2	89.67 (5)
N13^iv^—Cu2—N13	180.0	F3—Si1—F2	90.41 (5)
N13^iv^—Cu2—N19^v^	90.73 (5)	F4—Si1—F2	89.64 (4)
N13—Cu2—N19^v^	89.27 (5)	F6—Si1—F1	90.89 (4)
N13^iv^—Cu2—N19^vi^	89.27 (5)	F5—Si1—F1	90.35 (5)
N13—Cu2—N19^vi^	90.73 (5)	F3—Si1—F1	89.56 (5)
N19^v^—Cu2—N19^vi^	180.0	F4—Si1—F1	88.45 (4)
N13^iv^—Cu2—F2	90.59 (4)	F2—Si1—F1	178.09 (4)
N13—Cu2—F2	89.41 (4)	Si1—F1—Cu1	178.36 (5)

Symmetry codes: (i) 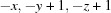; (ii) 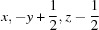; (iii) 
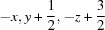
; (iv) 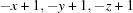; (v) 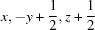; (vi) 
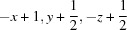
.

**Table 2:** Hydrogen-bond geometry (Å, °)

*D*—H⋯*A*	*D*—H	H⋯*A*	*D*⋯*A*	*D*—H⋯*A*
C17—H17⋯N31^vii^	0.95	2.34	3.2849 (19)	170
C20—H20⋯F2^ii^	0.95	2.32	3.0341 (16)	131
C20—H20⋯F5^ii^	0.95	2.53	3.4305 (17)	159
C18—H18⋯F2^iv^	0.95	2.52	3.1085 (16)	120
C9—H9⋯O52	0.95	2.50	3.4223 (18)	163
C3—H3⋯N37^viii^	0.95	2.54	3.4803 (19)	173
C21—H21⋯O52^ii^	0.95	2.56	3.4294 (18)	152
C14—H14⋯F2	0.95	2.43	3.0497 (16)	123
C14—H14⋯F4	0.95	2.55	3.4519 (17)	158
C8—H8⋯F1^v^	0.95	2.33	3.0205 (16)	129
C8—H8⋯F4^v^	0.95	2.51	3.3524 (18)	148
C5—H5⋯O52	0.95	2.43	3.2916 (18)	151
C24—H24⋯F2^vii^	0.95	2.29	2.9997 (16)	131
C24—H24⋯F3^vii^	0.95	2.55	3.4609 (17)	161
C11—H11⋯N37^viii^	0.95	2.61	3.4358 (19)	145
C12—H12⋯F1^viii^	0.95	2.26	2.9662 (16)	131
C12—H12⋯F6^viii^	0.95	2.49	3.4040 (16)	161
C2—H2⋯F1^i^	0.95	2.52	3.0708 (16)	117
C2—H2⋯F3^i^	0.95	2.45	3.3364 (17)	156
C45—H45⋯O50^ix^	0.95	2.44	3.376 (2)	167
C6—H6⋯F1	0.95	2.46	3.0691 (16)	122
C6—H6⋯F4	0.95	2.60	3.5313 (17)	166
C38—H38⋯F6	0.95	2.41	3.1240 (19)	132
C48—H48⋯O51^iv^	0.95	2.50	3.330 (2)	146
O53—H53*B*⋯F3^v^	0.78 (2)	1.96 (3)	2.7228 (17)	166 (2)
O52—H52*B*⋯O53	0.90 (3)	1.83 (3)	2.7227 (18)	170 (2)
O53—H53*A*⋯N43^iv^	0.85 (3)	2.05 (3)	2.8666 (19)	161 (3)
O52—H52*A*⋯O51	0.90 (3)	1.84 (3)	2.726 (2)	172 (2)
O50—H50*A*⋯O49	1.05 (3)	1.72 (3)	2.741 (2)	163 (2)
O49—H49*B*⋯F5^ii^	0.96 (3)	1.82 (3)	2.7716 (18)	173 (3)
O50—H50*B*⋯N25^i^	0.96 (3)	1.86 (3)	2.816 (2)	173 (3)
O49—H49*A*⋯O52^ii^	1.00 (4)	1.81 (4)	2.798 (2)	169 (3)
O51—H51*B*⋯F4	0.91 (3)	1.88 (3)	2.7029 (18)	149 (2)
O51—H51*A*⋯O50	1.00 (4)	1.76 (4)	2.719 (2)	158 (3)

Symmetry codes: (i) 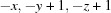; (ii) 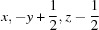; (iv) 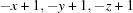; (v) 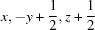; (vii) 
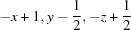
; (viii) 
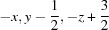
; (ix) 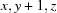.

**Table 3:** Geometrical parameters (Å, °) for the π–π stacking of the 4,4′-bi­pyridine mol­ecules within the pores *Cg*(*I*) is the centroid of the atoms defining plane *I*: *Cg*(N25) = N25/C26–C30; *Cg*(N31) = N31/C32–C36; *Cg*(N43) = N43/C44–7-C48 and *Cg*(N37) = N37/C38–C42. *d*_*Cg*–*Cg*_ is the distance between *Cg*(*I*) and *Cg*(*J*). α is the dihedral angle between planes *I* and *J*. β is the angle between the *Cg*(*I*)→*Cg*(*J*) vector and the normal to plane *I*. γ is the angle between the *Cg*(*I*)→*Cg*(*J*) vector and the normal to plane *J*.

*Cg*(*I*)	*Cg*(*J*)	*d*_*Cg*–*Cg*_	α	β	γ
N25	N25^i^	3.7374 (10)	0.02 (9)	9.2	9.2
N31	N37^ii^	3.7358 (9)	20.79 (8)	21.4	13.1
N43	N43^iii^	3.8576 (9)	0.00 (7)	23.8	23.8

Symmetry codes: (i) −*x*, 2 − *y*, 1 − *z*; (ii) *x*, *y*, *z*; (iii) 1 − *x*, 2 − *y*, 1 − *z*.

**Table 4:** Experimental details

Crystal data	
Chemical formula	[Cu(SiF_6_)(C_10_H_8_N_2_)_2_]·2C_10_H_8_N_2_·5H_2_O
*M*_r_	920.44
Crystal system, space group	Monoclinic, *P*2_1_/*c*
Temperature (K)	110
*a*, *b*, *c* (Å)	16.3875 (2), 16.6136 (2), 14.7959 (2)
β (°)	90.654 (1)
*V* (Å^3^)	4028.00 (9)
*Z*	4
Radiation type	Cu *K*α
μ (mm^−1^)	1.78
Crystal size (mm)	0.25 × 0.16 × 0.14

Data collection	
Diffractometer	Rigaku Oxford Diffraction SuperNova (Cu) X-ray Source
Absorption correction	Analytical [*CrysAlis PRO* (Rigaku Oxford Diffraction, 2015 ▸) based on expressions derived by Clark & Reid (1995 ▸)]
*T*_min_, *T*_max_	0.708, 0.824
No. of measured, independent and observed [*I* > 2σ(*I*)] reflections	68271, 8435, 7846
*R*_int_	0.029
(sin θ/λ)_max_ (Å^−1^)	0.630

Refinement	
*R*[*F* ^2^ > 2σ(*F* ^2^)], *wR*(*F* ^2^), *S*	0.036, 0.110, 1.06
No. of reflections	8435
No. of parameters	593
H-atom treatment	H atoms treated by a mixture of independent and constrained refinement
Δρ_max_, Δρ_min_ (e Å^−3^)	0.47, −0.50

Computer programs: *CrysAlis PRO* (Rigaku Oxford Diffraction, 2015 ▸), *SHELXT* (Sheldrick, 2015*a* ▸), *SHELXL* (Sheldrick, 2015*b* ▸), *Mercury* (Macrae *et al.*, 2006 ▸) and *publCIF* (Westrip, 2010 ▸).

## supplementary crystallographic information

### Crystal data

**Table:**

[Cu(SiF_6_)(C_10_H_8_N_2_)_2_]·2C_10_H_8_N_2_·5H_2_O	*F*(000) = 1900
*M**_r_* = 920.44	*D*_x_ = 1.518 Mg m^−^^3^
Monoclinic, *P*2_1_/*c*	Cu *K*α radiation, λ = 1.54184 Å
*a* = 16.3875 (2) Å	Cell parameters from 32121 reflections
*b* = 16.6136 (2) Å	θ = 3.7–76.5°
*c* = 14.7959 (2) Å	µ = 1.78 mm^−^^1^
β = 90.654 (1)°	*T* = 110 K
*V* = 4028.00 (9) Å^3^	Prism, violet
*Z* = 4	0.25 × 0.16 × 0.14 mm

### Data collection

**Table:**

Rigaku Oxford Diffraction SuperNova (Cu) X-ray Source diffractometer	7846 reflections with *I* > 2σ(*I*)
Radiation source: micro-focus sealed X-ray tube	*R*_int_ = 0.029
ω scans	θ_max_ = 76.2°, θ_min_ = 3.8°
Absorption correction: analytical [CrysAlis PRO (Rigaku Oxford Diffraction, 2015)]; analytical numeric absorption correction using a multi-faceted crystal model based on expressions derived by Clark & Reid (1995)	*h* = −20→20
*T*_min_ = 0.708, *T*_max_ = 0.824	*k* = 0→20
68271 measured reflections	*l* = 0→18
8435 independent reflections	

### Refinement

**Table:**

Refinement on *F*^2^	Primary atom site location: iterative
Least-squares matrix: full	Hydrogen site location: difference Fourier map
*R*[*F*^2^ > 2σ(*F*^2^)] = 0.036	H atoms treated by a mixture of independent and constrained refinement
*wR*(*F*^2^) = 0.110	*w* = 1/[σ^2^(*F*_o_^2^) + (0.0562*P*)^2^ + 2.6502*P*] where *P* = (*F*_o_^2^ + 2*F*_c_^2^)/3
*S* = 1.06	(Δ/σ)_max_ = 0.002
8435 reflections	Δρ_max_ = 0.47 e Å^−^^3^
593 parameters	Δρ_min_ = −0.50 e Å^−^^3^
0 restraints	

### Special details

**Table:**

Geometry. All esds (except the esd in the dihedral angle between two l.s. planes) are estimated using the full covariance matrix. The cell esds are taken into account individually in the estimation of esds in distances, angles and torsion angles; correlations between esds in cell parameters are only used when they are defined by crystal symmetry. An approximate (isotropic) treatment of cell esds is used for estimating esds involving l.s. planes.

### Fractional atomic coordinates and isotropic or equivalent isotropic displacement parameters (Å^2^)

**Table:**

	*x*	*y*	*z*	*U*_iso_*/*U*_eq_	
Cu1	0.0000	0.5000	0.5000	0.00740 (9)	
Cu2	0.5000	0.5000	0.5000	0.00808 (9)	
Si1	0.24851 (2)	0.50181 (2)	0.49951 (2)	0.01085 (11)	
F1	0.14392 (6)	0.50149 (4)	0.50157 (6)	0.01423 (19)	
F2	0.35288 (6)	0.49872 (4)	0.49705 (6)	0.01514 (19)	
F5	0.25214 (6)	0.49217 (7)	0.61255 (7)	0.0327 (3)	
F6	0.25001 (5)	0.60099 (6)	0.50857 (8)	0.0313 (3)	
F3	0.24470 (6)	0.51068 (7)	0.38628 (7)	0.0305 (2)	
F4	0.24575 (5)	0.40086 (6)	0.48849 (9)	0.0356 (3)	
N1	−0.00035 (7)	0.41543 (7)	0.59766 (8)	0.0104 (2)	
O52	0.24669 (7)	0.22533 (8)	0.69796 (10)	0.0304 (3)	
N7	−0.00003 (7)	0.09067 (7)	0.90635 (7)	0.0104 (2)	
N19	0.50157 (7)	0.08206 (7)	0.10342 (7)	0.0103 (2)	
N13	0.50009 (7)	0.40834 (7)	0.41061 (7)	0.0102 (2)	
O53	0.29213 (8)	0.07136 (8)	0.73649 (9)	0.0319 (3)	
O50	0.27901 (8)	0.11159 (8)	0.42810 (9)	0.0326 (3)	
O51	0.29696 (9)	0.24956 (9)	0.52512 (10)	0.0374 (3)	
N43	0.55301 (8)	0.94138 (8)	0.35053 (9)	0.0224 (3)	
O49	0.22422 (9)	0.11500 (9)	0.25239 (9)	0.0375 (3)	
N37	0.23466 (8)	0.82015 (8)	0.64527 (10)	0.0244 (3)	
N31	0.26526 (8)	0.82439 (10)	0.32164 (10)	0.0281 (3)	
C16	0.50172 (8)	0.27683 (8)	0.29304 (9)	0.0117 (3)	
C17	0.56598 (8)	0.33166 (8)	0.29579 (9)	0.0136 (3)	
H17	0.6114	0.3250	0.2572	0.016*	
C22	0.50242 (8)	0.20746 (8)	0.23027 (9)	0.0113 (3)	
C4	−0.00106 (8)	0.28967 (8)	0.72282 (9)	0.0131 (3)	
C20	0.43162 (8)	0.11255 (8)	0.13529 (9)	0.0141 (3)	
H20	0.3814	0.0907	0.1139	0.017*	
C10	−0.00090 (8)	0.22170 (8)	0.78738 (9)	0.0132 (3)	
C15	0.43615 (8)	0.29027 (8)	0.35038 (9)	0.0146 (3)	
H15	0.3911	0.2543	0.3504	0.018*	
C18	0.56320 (8)	0.39594 (8)	0.35523 (9)	0.0125 (3)	
H18	0.6076	0.4327	0.3569	0.015*	
C9	0.07202 (8)	0.18464 (9)	0.81404 (10)	0.0164 (3)	
H9	0.1227	0.2038	0.7920	0.020*	
C3	−0.06460 (8)	0.34570 (8)	0.71891 (9)	0.0144 (3)	
H3	−0.1089	0.3418	0.7595	0.017*	
C21	0.42956 (8)	0.17432 (9)	0.19781 (9)	0.0145 (3)	
H21	0.3787	0.1941	0.2186	0.017*	
C14	0.43721 (8)	0.35642 (8)	0.40738 (9)	0.0147 (3)	
H14	0.3919	0.3654	0.4455	0.018*	
C8	0.06999 (8)	0.11997 (9)	0.87270 (10)	0.0155 (3)	
H8	0.1200	0.0952	0.8900	0.019*	
C5	0.06262 (8)	0.29879 (9)	0.66202 (10)	0.0162 (3)	
H5	0.1066	0.2616	0.6620	0.019*	
C24	0.57206 (8)	0.11186 (9)	0.13636 (10)	0.0156 (3)	
H24	0.6220	0.0897	0.1158	0.019*	
C23	0.57484 (8)	0.17385 (9)	0.19922 (10)	0.0160 (3)	
H23	0.6259	0.1933	0.2210	0.019*	
C11	−0.07321 (8)	0.19167 (9)	0.82304 (9)	0.0147 (3)	
H11	−0.1240	0.2156	0.8072	0.018*	
C12	−0.07035 (8)	0.12663 (9)	0.88184 (9)	0.0142 (3)	
H12	−0.1200	0.1068	0.9057	0.017*	
C2	−0.06252 (8)	0.40681 (8)	0.65549 (9)	0.0130 (3)	
H2	−0.1065	0.4440	0.6527	0.016*	
C45	0.42153 (9)	0.96535 (9)	0.41679 (11)	0.0208 (3)	
H45	0.3752	0.9994	0.4215	0.025*	
C6	0.06131 (8)	0.36237 (9)	0.60178 (10)	0.0152 (3)	
H6	0.1058	0.3687	0.5618	0.018*	
C46	0.42614 (9)	0.89392 (9)	0.46622 (10)	0.0173 (3)	
C44	0.48547 (10)	0.98589 (10)	0.36069 (11)	0.0226 (3)	
H44	0.4811	1.0346	0.3273	0.027*	
C47	0.49637 (9)	0.84765 (9)	0.45646 (10)	0.0201 (3)	
H47	0.5025	0.7986	0.4889	0.024*	
C38	0.28158 (9)	0.76657 (9)	0.60272 (10)	0.0208 (3)	
H38	0.2714	0.7110	0.6125	0.025*	
N25	−0.12407 (9)	0.91210 (10)	0.49597 (11)	0.0340 (4)	
C39	0.34424 (9)	0.78762 (9)	0.54526 (10)	0.0195 (3)	
H39	0.3764	0.7470	0.5179	0.023*	
C40	0.36000 (9)	0.86869 (9)	0.52772 (10)	0.0180 (3)	
C36	0.22422 (10)	0.77389 (11)	0.37480 (11)	0.0253 (3)	
H36	0.2459	0.7215	0.3846	0.030*	
C48	0.55719 (9)	0.87357 (10)	0.39917 (11)	0.0222 (3)	
H48	0.6047	0.8413	0.3941	0.027*	
C34	0.11765 (9)	0.86961 (10)	0.40037 (10)	0.0224 (3)	
C35	0.15162 (9)	0.79402 (10)	0.41632 (11)	0.0231 (3)	
H35	0.1254	0.7568	0.4551	0.028*	
C33	0.16047 (10)	0.92295 (10)	0.34582 (11)	0.0266 (3)	
H33	0.1399	0.9754	0.3338	0.032*	
C28	0.03559 (10)	0.88875 (10)	0.43575 (11)	0.0232 (3)	
C41	0.31092 (10)	0.92455 (10)	0.57120 (12)	0.0254 (3)	
H41	0.3184	0.9805	0.5613	0.030*	
C27	0.01285 (10)	0.86650 (11)	0.52287 (12)	0.0286 (4)	
H27	0.0513	0.8423	0.5630	0.034*	
C32	0.23375 (10)	0.89772 (11)	0.30952 (12)	0.0286 (4)	
H32	0.2634	0.9349	0.2738	0.034*	
C42	0.25108 (10)	0.89764 (10)	0.62910 (13)	0.0299 (4)	
H42	0.2195	0.9370	0.6595	0.036*	
C26	−0.06647 (11)	0.88027 (12)	0.54980 (13)	0.0335 (4)	
H26	−0.0808	0.8663	0.6098	0.040*	
C29	−0.02335 (11)	0.92411 (11)	0.38086 (12)	0.0302 (4)	
H29	−0.0100	0.9422	0.3219	0.036*	
C30	−0.10216 (12)	0.93284 (12)	0.41284 (14)	0.0367 (4)	
H30	−0.1425	0.9547	0.3734	0.044*	
H53B	0.2849 (14)	0.0514 (14)	0.7835 (17)	0.034 (6)*	
H52B	0.2595 (15)	0.1754 (16)	0.7175 (17)	0.046 (7)*	
H53A	0.3396 (18)	0.0590 (18)	0.720 (2)	0.066 (9)*	
H52A	0.2588 (15)	0.2311 (15)	0.6395 (18)	0.046 (7)*	
H50A	0.2641 (16)	0.1230 (17)	0.360 (2)	0.060 (8)*	
H49B	0.2334 (18)	0.0814 (19)	0.201 (2)	0.069 (9)*	
H50B	0.2281 (19)	0.0997 (19)	0.457 (2)	0.072 (9)*	
H49A	0.234 (2)	0.174 (2)	0.241 (2)	0.100 (12)*	
H51B	0.2650 (13)	0.2899 (15)	0.5027 (14)	0.034 (6)*	
H51A	0.281 (2)	0.207 (2)	0.480 (2)	0.093 (11)*	

### Atomic displacement parameters (Å^2^)

**Table:**

	*U*^11^	*U*^22^	*U*^33^	*U*^12^	*U*^13^	*U*^23^
Cu1	0.01176 (16)	0.00446 (15)	0.00599 (15)	−0.00002 (9)	0.00097 (11)	0.00010 (8)
Cu2	0.01341 (16)	0.00456 (15)	0.00628 (15)	0.00000 (9)	0.00100 (11)	−0.00001 (8)
Si1	0.0105 (2)	0.0103 (2)	0.0118 (2)	0.00012 (11)	0.00098 (16)	−0.00039 (12)
F1	0.0130 (4)	0.0140 (5)	0.0157 (4)	−0.0002 (3)	0.0016 (3)	−0.0021 (3)
F2	0.0138 (4)	0.0154 (5)	0.0162 (5)	0.0010 (3)	0.0015 (3)	0.0020 (3)
F5	0.0194 (5)	0.0653 (8)	0.0135 (5)	−0.0001 (4)	0.0007 (4)	0.0059 (4)
F6	0.0196 (5)	0.0126 (5)	0.0616 (7)	−0.0008 (3)	0.0034 (5)	−0.0057 (4)
F3	0.0187 (5)	0.0589 (7)	0.0139 (5)	0.0055 (4)	0.0012 (4)	−0.0002 (4)
F4	0.0198 (5)	0.0123 (5)	0.0746 (8)	0.0007 (3)	0.0011 (5)	−0.0033 (4)
N1	0.0130 (5)	0.0087 (5)	0.0096 (5)	−0.0006 (4)	0.0001 (4)	0.0019 (4)
O52	0.0243 (6)	0.0258 (6)	0.0412 (8)	0.0045 (5)	0.0085 (5)	0.0034 (5)
N7	0.0137 (5)	0.0086 (5)	0.0088 (5)	−0.0003 (4)	0.0007 (4)	0.0019 (4)
N19	0.0145 (5)	0.0079 (5)	0.0085 (5)	0.0004 (4)	0.0004 (4)	−0.0010 (4)
N13	0.0139 (5)	0.0072 (5)	0.0094 (5)	0.0003 (4)	0.0005 (4)	−0.0011 (4)
O53	0.0346 (7)	0.0311 (7)	0.0302 (7)	0.0059 (5)	0.0124 (5)	0.0096 (5)
O50	0.0271 (6)	0.0342 (7)	0.0366 (7)	0.0047 (5)	0.0013 (5)	−0.0021 (5)
O51	0.0474 (8)	0.0258 (7)	0.0389 (7)	0.0018 (6)	−0.0021 (6)	0.0054 (6)
N43	0.0228 (6)	0.0233 (7)	0.0212 (6)	−0.0028 (5)	0.0009 (5)	−0.0016 (5)
O49	0.0472 (8)	0.0342 (7)	0.0310 (7)	0.0016 (6)	0.0001 (6)	0.0007 (6)
N37	0.0198 (6)	0.0231 (7)	0.0305 (7)	−0.0016 (5)	0.0044 (5)	−0.0003 (6)
N31	0.0206 (6)	0.0401 (8)	0.0238 (7)	−0.0031 (6)	0.0021 (5)	−0.0034 (6)
C16	0.0136 (6)	0.0099 (6)	0.0118 (6)	0.0007 (5)	−0.0003 (5)	−0.0022 (5)
C17	0.0134 (6)	0.0136 (6)	0.0138 (6)	−0.0008 (5)	0.0031 (5)	−0.0031 (5)
C22	0.0142 (6)	0.0089 (6)	0.0109 (6)	−0.0007 (5)	0.0007 (5)	−0.0015 (5)
C4	0.0139 (6)	0.0118 (6)	0.0137 (6)	−0.0001 (5)	0.0003 (5)	0.0042 (5)
C20	0.0133 (6)	0.0127 (6)	0.0162 (7)	−0.0014 (5)	0.0012 (5)	−0.0040 (5)
C10	0.0148 (6)	0.0116 (6)	0.0132 (6)	0.0004 (5)	0.0016 (5)	0.0047 (5)
C15	0.0143 (6)	0.0126 (6)	0.0171 (7)	−0.0030 (5)	0.0032 (5)	−0.0043 (5)
C18	0.0140 (6)	0.0113 (6)	0.0122 (6)	−0.0010 (5)	0.0006 (5)	−0.0018 (5)
C9	0.0121 (6)	0.0179 (7)	0.0193 (7)	−0.0003 (5)	0.0022 (5)	0.0080 (6)
C3	0.0141 (6)	0.0155 (7)	0.0136 (6)	0.0015 (5)	0.0032 (5)	0.0042 (5)
C21	0.0126 (6)	0.0145 (7)	0.0164 (7)	0.0000 (5)	0.0022 (5)	−0.0045 (5)
C14	0.0159 (6)	0.0126 (6)	0.0157 (6)	−0.0011 (5)	0.0037 (5)	−0.0035 (5)
C8	0.0139 (6)	0.0148 (7)	0.0179 (7)	0.0016 (5)	0.0015 (5)	0.0058 (5)
C5	0.0131 (6)	0.0151 (7)	0.0205 (7)	0.0028 (5)	0.0032 (5)	0.0071 (5)
C24	0.0136 (6)	0.0154 (7)	0.0177 (7)	0.0016 (5)	0.0009 (5)	−0.0061 (5)
C23	0.0125 (6)	0.0175 (7)	0.0179 (7)	−0.0002 (5)	−0.0012 (5)	−0.0067 (5)
C11	0.0119 (6)	0.0153 (7)	0.0168 (6)	0.0016 (5)	0.0009 (5)	0.0057 (5)
C12	0.0132 (6)	0.0148 (7)	0.0147 (6)	−0.0005 (5)	0.0008 (5)	0.0044 (5)
C2	0.0145 (6)	0.0128 (6)	0.0118 (6)	0.0018 (5)	0.0005 (5)	0.0018 (5)
C45	0.0192 (7)	0.0177 (7)	0.0255 (8)	0.0006 (6)	−0.0016 (6)	−0.0001 (6)
C6	0.0139 (6)	0.0145 (7)	0.0174 (7)	0.0014 (5)	0.0032 (5)	0.0058 (5)
C46	0.0176 (7)	0.0174 (7)	0.0169 (7)	−0.0015 (5)	−0.0028 (5)	−0.0021 (5)
C44	0.0252 (8)	0.0187 (7)	0.0237 (8)	−0.0015 (6)	−0.0007 (6)	0.0022 (6)
C47	0.0205 (7)	0.0192 (7)	0.0205 (7)	0.0017 (6)	−0.0016 (6)	0.0003 (6)
C38	0.0222 (7)	0.0182 (7)	0.0219 (7)	−0.0019 (6)	−0.0013 (6)	0.0005 (6)
N25	0.0270 (7)	0.0306 (8)	0.0446 (9)	0.0051 (6)	0.0069 (7)	−0.0021 (7)
C39	0.0228 (7)	0.0171 (7)	0.0186 (7)	0.0014 (6)	−0.0001 (6)	−0.0008 (6)
C40	0.0158 (6)	0.0190 (7)	0.0193 (7)	−0.0011 (5)	−0.0024 (5)	0.0000 (6)
C36	0.0198 (7)	0.0319 (9)	0.0241 (8)	0.0008 (6)	−0.0012 (6)	−0.0023 (7)
C48	0.0194 (7)	0.0242 (8)	0.0230 (8)	0.0018 (6)	0.0002 (6)	−0.0020 (6)
C34	0.0214 (7)	0.0263 (8)	0.0194 (7)	−0.0025 (6)	0.0009 (6)	−0.0037 (6)
C35	0.0203 (7)	0.0283 (8)	0.0205 (7)	−0.0026 (6)	−0.0004 (6)	0.0006 (6)
C33	0.0294 (8)	0.0249 (8)	0.0255 (8)	−0.0039 (6)	0.0021 (6)	−0.0007 (6)
C28	0.0239 (8)	0.0210 (8)	0.0247 (8)	−0.0023 (6)	0.0024 (6)	−0.0037 (6)
C41	0.0216 (7)	0.0160 (7)	0.0387 (9)	−0.0005 (6)	0.0060 (7)	−0.0003 (6)
C27	0.0259 (8)	0.0352 (9)	0.0246 (8)	0.0020 (7)	0.0019 (6)	0.0002 (7)
C32	0.0260 (8)	0.0347 (9)	0.0253 (8)	−0.0092 (7)	0.0043 (6)	−0.0018 (7)
C42	0.0239 (8)	0.0204 (8)	0.0455 (10)	0.0011 (6)	0.0122 (7)	−0.0028 (7)
C26	0.0317 (9)	0.0346 (10)	0.0343 (10)	0.0012 (7)	0.0113 (7)	0.0000 (8)
C29	0.0334 (9)	0.0264 (9)	0.0308 (9)	0.0047 (7)	0.0041 (7)	0.0029 (7)
C30	0.0318 (9)	0.0341 (10)	0.0442 (11)	0.0112 (8)	0.0010 (8)	0.0038 (8)

### Geometric parameters (Å, º)

**Table:**

Cu1—N1^i^	2.0156 (11)	C10—C9	1.3971 (19)
Cu1—N1	2.0156 (11)	C15—C14	1.3852 (19)
Cu1—N7^ii^	2.0467 (11)	C15—H15	0.9500
Cu1—N7^iii^	2.0467 (11)	C18—H18	0.9500
Cu1—F1^i^	2.3585 (9)	C9—C8	1.3818 (19)
Cu1—F1	2.3585 (9)	C9—H9	0.9500
Cu2—N13^iv^	2.0170 (11)	C3—C2	1.3832 (19)
Cu2—N13	2.0170 (11)	C3—H3	0.9500
Cu2—N19^v^	2.0494 (11)	C21—H21	0.9500
Cu2—N19^vi^	2.0494 (11)	C14—H14	0.9500
Cu2—F2	2.4109 (9)	C8—H8	0.9500
Cu2—F2^iv^	2.4109 (9)	C5—C6	1.3821 (19)
Si1—F6	1.6534 (11)	C5—H5	0.9500
Si1—F5	1.6806 (10)	C24—C23	1.3882 (19)
Si1—F3	1.6823 (10)	C24—H24	0.9500
Si1—F4	1.6855 (11)	C23—H23	0.9500
Si1—F2	1.7120 (10)	C11—C12	1.3877 (19)
Si1—F1	1.7145 (10)	C11—H11	0.9500
N1—C6	1.3418 (18)	C12—H12	0.9500
N1—C2	1.3455 (17)	C2—H2	0.9500
O52—H52B	0.90 (3)	C45—C44	1.387 (2)
O52—H52A	0.90 (3)	C45—C46	1.396 (2)
N7—C12	1.3442 (18)	C45—H45	0.9500
N7—C8	1.3470 (18)	C6—H6	0.9500
N7—Cu1^vii^	2.0467 (11)	C46—C47	1.393 (2)
N19—C24	1.3432 (18)	C46—C40	1.484 (2)
N19—C20	1.3436 (18)	C44—H44	0.9500
N19—Cu2^viii^	2.0494 (11)	C47—C48	1.384 (2)
N13—C18	1.3426 (17)	C47—H47	0.9500
N13—C14	1.3443 (18)	C38—C39	1.385 (2)
O53—H53B	0.78 (2)	C38—H38	0.9500
O53—H53A	0.85 (3)	N25—C30	1.331 (3)
O50—H50A	1.05 (3)	N25—C26	1.337 (3)
O50—H50B	0.96 (3)	C39—C40	1.396 (2)
O51—H51B	0.91 (3)	C39—H39	0.9500
O51—H51A	1.00 (4)	C40—C41	1.391 (2)
N43—C48	1.338 (2)	C36—C35	1.386 (2)
N43—C44	1.341 (2)	C36—H36	0.9500
O49—H49B	0.96 (3)	C48—H48	0.9500
O49—H49A	1.00 (4)	C34—C35	1.393 (2)
N37—C42	1.337 (2)	C34—C33	1.394 (2)
N37—C38	1.338 (2)	C34—C28	1.483 (2)
N31—C32	1.334 (2)	C35—H35	0.9500
N31—C36	1.337 (2)	C33—C32	1.386 (2)
C16—C17	1.3927 (19)	C33—H33	0.9500
C16—C15	1.3947 (18)	C28—C29	1.386 (2)
C16—C22	1.4803 (19)	C28—C27	1.396 (2)
C17—C18	1.3845 (19)	C41—C42	1.384 (2)
C17—H17	0.9500	C41—H41	0.9500
C22—C23	1.3942 (19)	C27—C26	1.383 (2)
C22—C21	1.3950 (19)	C27—H27	0.9500
C4—C5	1.3938 (19)	C32—H32	0.9500
C4—C3	1.3974 (19)	C42—H42	0.9500
C4—C10	1.4791 (19)	C26—H26	0.9500
C20—C21	1.3823 (19)	C29—C30	1.388 (3)
C20—H20	0.9500	C29—H29	0.9500
C10—C11	1.3948 (19)	C30—H30	0.9500
			
N1^i^—Cu1—N1	180.00 (6)	C2—C3—C4	119.53 (12)
N1^i^—Cu1—N7^ii^	91.58 (5)	C2—C3—H3	120.2
N1—Cu1—N7^ii^	88.42 (5)	C4—C3—H3	120.2
N1^i^—Cu1—N7^iii^	88.42 (5)	C20—C21—C22	119.74 (12)
N1—Cu1—N7^iii^	91.58 (5)	C20—C21—H21	120.1
N7^ii^—Cu1—N7^iii^	180.0	C22—C21—H21	120.1
N1^i^—Cu1—F1^i^	90.65 (4)	N13—C14—C15	122.35 (12)
N1—Cu1—F1^i^	89.35 (4)	N13—C14—H14	118.8
N7^ii^—Cu1—F1^i^	89.60 (4)	C15—C14—H14	118.8
N7^iii^—Cu1—F1^i^	90.40 (4)	N7—C8—C9	122.68 (13)
N1^i^—Cu1—F1	89.35 (4)	N7—C8—H8	118.7
N1—Cu1—F1	90.65 (4)	C9—C8—H8	118.7
N7^ii^—Cu1—F1	90.40 (4)	C6—C5—C4	119.54 (13)
N7^iii^—Cu1—F1	89.60 (4)	C6—C5—H5	120.2
F1^i^—Cu1—F1	180.0	C4—C5—H5	120.2
N13^iv^—Cu2—N13	180.0	N19—C24—C23	122.55 (12)
N13^iv^—Cu2—N19^v^	90.73 (5)	N19—C24—H24	118.7
N13—Cu2—N19^v^	89.27 (5)	C23—C24—H24	118.7
N13^iv^—Cu2—N19^vi^	89.27 (5)	C24—C23—C22	119.76 (12)
N13—Cu2—N19^vi^	90.73 (5)	C24—C23—H23	120.1
N19^v^—Cu2—N19^vi^	180.0	C22—C23—H23	120.1
N13^iv^—Cu2—F2	90.59 (4)	C12—C11—C10	119.54 (12)
N13—Cu2—F2	89.41 (4)	C12—C11—H11	120.2
N19^v^—Cu2—F2	90.67 (4)	C10—C11—H11	120.2
N19^vi^—Cu2—F2	89.33 (4)	N7—C12—C11	122.54 (12)
N13^iv^—Cu2—F2^iv^	89.41 (4)	N7—C12—H12	118.7
N13—Cu2—F2^iv^	90.59 (4)	C11—C12—H12	118.7
N19^v^—Cu2—F2^iv^	89.33 (4)	N1—C2—C3	122.29 (12)
N19^vi^—Cu2—F2^iv^	90.67 (4)	N1—C2—H2	118.9
F2—Cu2—F2^iv^	180.0	C3—C2—H2	118.9
F6—Si1—F5	90.80 (6)	C44—C45—C46	119.09 (14)
F6—Si1—F3	89.64 (6)	C44—C45—H45	120.5
F5—Si1—F3	179.55 (6)	C46—C45—H45	120.5
F6—Si1—F4	178.87 (6)	N1—C6—C5	122.51 (12)
F5—Si1—F4	90.12 (6)	N1—C6—H6	118.7
F3—Si1—F4	89.44 (6)	C5—C6—H6	118.7
F6—Si1—F2	91.01 (4)	C47—C46—C45	117.06 (14)
F5—Si1—F2	89.67 (5)	C47—C46—C40	121.19 (14)
F3—Si1—F2	90.41 (5)	C45—C46—C40	121.74 (13)
F4—Si1—F2	89.64 (4)	N43—C44—C45	124.17 (15)
F6—Si1—F1	90.89 (4)	N43—C44—H44	117.9
F5—Si1—F1	90.35 (5)	C45—C44—H44	117.9
F3—Si1—F1	89.56 (5)	C48—C47—C46	119.55 (14)
F4—Si1—F1	88.45 (4)	C48—C47—H47	120.2
F2—Si1—F1	178.09 (4)	C46—C47—H47	120.2
Si1—F1—Cu1	178.36 (5)	N37—C38—C39	123.69 (14)
Si1—F2—Cu2	176.83 (5)	N37—C38—H38	118.2
C6—N1—C2	118.46 (12)	C39—C38—H38	118.2
C6—N1—Cu1	118.82 (9)	C30—N25—C26	117.18 (15)
C2—N1—Cu1	122.58 (9)	C38—C39—C40	119.83 (14)
H52B—O52—H52A	111 (2)	C38—C39—H39	120.1
C12—N7—C8	118.09 (12)	C40—C39—H39	120.1
C12—N7—Cu1^vii^	120.22 (9)	C41—C40—C39	116.65 (14)
C8—N7—Cu1^vii^	121.50 (9)	C41—C40—C46	121.72 (14)
C24—N19—C20	117.86 (12)	C39—C40—C46	121.64 (13)
C24—N19—Cu2^viii^	121.30 (9)	N31—C36—C35	123.33 (16)
C20—N19—Cu2^viii^	120.62 (9)	N31—C36—H36	118.3
C18—N13—C14	118.38 (12)	C35—C36—H36	118.3
C18—N13—Cu2	121.49 (9)	N43—C48—C47	123.95 (14)
C14—N13—Cu2	120.10 (9)	N43—C48—H48	118.0
H53B—O53—H53A	108 (3)	C47—C48—H48	118.0
H50A—O50—H50B	105 (2)	C35—C34—C33	117.94 (15)
H51B—O51—H51A	98 (2)	C35—C34—C28	119.72 (14)
C48—N43—C44	116.18 (14)	C33—C34—C28	122.19 (15)
H49B—O49—H49A	114 (3)	C36—C35—C34	119.07 (15)
C42—N37—C38	115.99 (14)	C36—C35—H35	120.5
C32—N31—C36	117.09 (14)	C34—C35—H35	120.5
C17—C16—C15	117.72 (12)	C32—C33—C34	118.45 (16)
C17—C16—C22	121.09 (12)	C32—C33—H33	120.8
C15—C16—C22	121.19 (12)	C34—C33—H33	120.8
C18—C17—C16	119.51 (12)	C29—C28—C27	117.51 (15)
C18—C17—H17	120.2	C29—C28—C34	120.85 (15)
C16—C17—H17	120.2	C27—C28—C34	121.46 (15)
C23—C22—C21	117.19 (12)	C42—C41—C40	119.25 (15)
C23—C22—C16	122.08 (12)	C42—C41—H41	120.4
C21—C22—C16	120.70 (12)	C40—C41—H41	120.4
C5—C4—C3	117.65 (12)	C26—C27—C28	118.95 (17)
C5—C4—C10	120.26 (13)	C26—C27—H27	120.5
C3—C4—C10	122.08 (12)	C28—C27—H27	120.5
N19—C20—C21	122.84 (13)	N31—C32—C33	124.04 (16)
N19—C20—H20	118.6	N31—C32—H32	118.0
C21—C20—H20	118.6	C33—C32—H32	118.0
C11—C10—C9	117.59 (12)	N37—C42—C41	124.56 (15)
C11—C10—C4	121.48 (13)	N37—C42—H42	117.7
C9—C10—C4	120.93 (12)	C41—C42—H42	117.7
C14—C15—C16	119.54 (13)	N25—C26—C27	123.59 (17)
C14—C15—H15	120.2	N25—C26—H26	118.2
C16—C15—H15	120.2	C27—C26—H26	118.2
N13—C18—C17	122.50 (12)	C28—C29—C30	119.30 (17)
N13—C18—H18	118.8	C28—C29—H29	120.3
C17—C18—H18	118.8	C30—C29—H29	120.3
C8—C9—C10	119.56 (13)	N25—C30—C29	123.38 (18)
C8—C9—H9	120.2	N25—C30—H30	118.3
C10—C9—H9	120.2	C29—C30—H30	118.3
			
C15—C16—C17—C18	0.9 (2)	C4—C3—C2—N1	−1.1 (2)
C22—C16—C17—C18	179.87 (12)	C2—N1—C6—C5	1.0 (2)
C17—C16—C22—C23	26.6 (2)	Cu1—N1—C6—C5	−174.86 (11)
C15—C16—C22—C23	−154.49 (14)	C4—C5—C6—N1	−1.6 (2)
C17—C16—C22—C21	−151.72 (14)	C44—C45—C46—C47	0.7 (2)
C15—C16—C22—C21	27.22 (19)	C44—C45—C46—C40	179.73 (14)
C24—N19—C20—C21	2.0 (2)	C48—N43—C44—C45	−0.5 (2)
Cu2^viii^—N19—C20—C21	−172.69 (11)	C46—C45—C44—N43	−0.3 (2)
C5—C4—C10—C11	−154.60 (14)	C45—C46—C47—C48	−0.3 (2)
C3—C4—C10—C11	24.2 (2)	C40—C46—C47—C48	−179.31 (14)
C5—C4—C10—C9	24.7 (2)	C42—N37—C38—C39	0.3 (2)
C3—C4—C10—C9	−156.55 (14)	N37—C38—C39—C40	−1.4 (2)
C17—C16—C15—C14	−0.2 (2)	C38—C39—C40—C41	0.7 (2)
C22—C16—C15—C14	−179.17 (13)	C38—C39—C40—C46	−179.63 (13)
C14—N13—C18—C17	−0.5 (2)	C47—C46—C40—C41	149.49 (16)
Cu2—N13—C18—C17	177.47 (10)	C45—C46—C40—C41	−29.5 (2)
C16—C17—C18—N13	−0.6 (2)	C47—C46—C40—C39	−30.1 (2)
C11—C10—C9—C8	0.9 (2)	C45—C46—C40—C39	150.90 (15)
C4—C10—C9—C8	−178.38 (14)	C32—N31—C36—C35	0.5 (2)
C5—C4—C3—C2	0.5 (2)	C44—N43—C48—C47	1.0 (2)
C10—C4—C3—C2	−178.27 (13)	C46—C47—C48—N43	−0.6 (2)
N19—C20—C21—C22	0.0 (2)	N31—C36—C35—C34	2.0 (2)
C23—C22—C21—C20	−2.0 (2)	C33—C34—C35—C36	−2.6 (2)
C16—C22—C21—C20	176.33 (13)	C28—C34—C35—C36	173.10 (15)
C18—N13—C14—C15	1.2 (2)	C35—C34—C33—C32	0.8 (2)
Cu2—N13—C14—C15	−176.76 (11)	C28—C34—C33—C32	−174.74 (15)
C16—C15—C14—N13	−0.9 (2)	C35—C34—C28—C29	−132.33 (17)
C12—N7—C8—C9	−0.4 (2)	C33—C34—C28—C29	43.2 (2)
Cu1^vii^—N7—C8—C9	−175.57 (11)	C35—C34—C28—C27	42.8 (2)
C10—C9—C8—N7	−0.4 (2)	C33—C34—C28—C27	−141.73 (17)
C3—C4—C5—C6	0.8 (2)	C39—C40—C41—C42	0.8 (2)
C10—C4—C5—C6	179.62 (13)	C46—C40—C41—C42	−178.85 (15)
C20—N19—C24—C23	−2.0 (2)	C29—C28—C27—C26	0.3 (3)
Cu2^viii^—N19—C24—C23	172.69 (11)	C34—C28—C27—C26	−174.98 (16)
N19—C24—C23—C22	−0.1 (2)	C36—N31—C32—C33	−2.4 (3)
C21—C22—C23—C24	2.1 (2)	C34—C33—C32—N31	1.8 (3)
C16—C22—C23—C24	−176.27 (13)	C38—N37—C42—C41	1.3 (3)
C9—C10—C11—C12	−0.8 (2)	C40—C41—C42—N37	−1.9 (3)
C4—C10—C11—C12	178.56 (13)	C30—N25—C26—C27	−1.5 (3)
C8—N7—C12—C11	0.6 (2)	C28—C27—C26—N25	1.8 (3)
Cu1^vii^—N7—C12—C11	175.83 (11)	C27—C28—C29—C30	−2.6 (3)
C10—C11—C12—N7	0.0 (2)	C34—C28—C29—C30	172.72 (17)
C6—N1—C2—C3	0.4 (2)	C26—N25—C30—C29	−1.0 (3)
Cu1—N1—C2—C3	176.11 (10)	C28—C29—C30—N25	3.1 (3)

Symmetry codes: (i) −*x*, −*y*+1, −*z*+1; (ii) *x*, −*y*+1/2, *z*−1/2; (iii) −*x*, *y*+1/2, −*z*+3/2; (iv) −*x*+1, −*y*+1, −*z*+1; (v) *x*, −*y*+1/2, *z*+1/2; (vi) −*x*+1, *y*+1/2, −*z*+1/2; (vii) −*x*, *y*−1/2, −*z*+3/2; (viii) −*x*+1, *y*−1/2, −*z*+1/2.

### Hydrogen-bond geometry (Å, º)

**Table:**

*D*—H···*A*	*D*—H	H···*A*	*D*···*A*	*D*—H···*A*
C17—H17···N31^viii^	0.95	2.34	3.2849 (19)	170
C20—H20···F2^ii^	0.95	2.32	3.0341 (16)	131
C20—H20···F5^ii^	0.95	2.53	3.4305 (17)	159
C18—H18···F2^iv^	0.95	2.52	3.1085 (16)	120
C9—H9···O52	0.95	2.50	3.4223 (18)	163
C3—H3···N37^vii^	0.95	2.54	3.4803 (19)	173
C21—H21···O52^ii^	0.95	2.56	3.4294 (18)	152
C14—H14···F2	0.95	2.43	3.0497 (16)	123
C14—H14···F4	0.95	2.55	3.4519 (17)	158
C8—H8···F1^v^	0.95	2.33	3.0205 (16)	129
C8—H8···F4^v^	0.95	2.51	3.3524 (18)	148
C5—H5···O52	0.95	2.43	3.2916 (18)	151
C24—H24···F2^viii^	0.95	2.29	2.9997 (16)	131
C24—H24···F3^viii^	0.95	2.55	3.4609 (17)	161
C11—H11···N37^vii^	0.95	2.61	3.4358 (19)	145
C12—H12···F1^vii^	0.95	2.26	2.9662 (16)	131
C12—H12···F6^vii^	0.95	2.49	3.4040 (16)	161
C2—H2···F1^i^	0.95	2.52	3.0708 (16)	117
C2—H2···F3^i^	0.95	2.45	3.3364 (17)	156
C45—H45···O50^ix^	0.95	2.44	3.376 (2)	167
C6—H6···F1	0.95	2.46	3.0691 (16)	122
C6—H6···F4	0.95	2.60	3.5313 (17)	166
C38—H38···F6	0.95	2.41	3.1240 (19)	132
C48—H48···O51^iv^	0.95	2.50	3.330 (2)	146
O53—H53*B*···F3^v^	0.78 (2)	1.96 (3)	2.7228 (17)	166 (2)
O52—H52*B*···O53	0.90 (3)	1.83 (3)	2.7227 (18)	170 (2)
O53—H53*A*···N43^iv^	0.85 (3)	2.05 (3)	2.8666 (19)	161 (3)
O52—H52*A*···O51	0.90 (3)	1.84 (3)	2.726 (2)	172 (2)
O50—H50*A*···O49	1.05 (3)	1.72 (3)	2.741 (2)	163 (2)
O49—H49*B*···F5^ii^	0.96 (3)	1.82 (3)	2.7716 (18)	173 (3)
O50—H50*B*···N25^i^	0.96 (3)	1.86 (3)	2.816 (2)	173 (3)
O49—H49*A*···O52^ii^	1.00 (4)	1.81 (4)	2.798 (2)	169 (3)
O51—H51*B*···F4	0.91 (3)	1.88 (3)	2.7029 (18)	149 (2)
O51—H51*A*···O50	1.00 (4)	1.76 (4)	2.719 (2)	158 (3)

Symmetry codes: (i) −*x*, −*y*+1, −*z*+1; (ii) *x*, −*y*+1/2, *z*−1/2; (iv) −*x*+1, −*y*+1, −*z*+1; (v) *x*, −*y*+1/2, *z*+1/2; (vii) −*x*, *y*−1/2, −*z*+3/2; (viii) −*x*+1, *y*−1/2, −*z*+1/2; (ix) *x*, *y*+1, *z*.
